# Sarcoidosis: Causes, Diagnosis, Clinical Features, and Treatments

**DOI:** 10.3390/jcm9041081

**Published:** 2020-04-10

**Authors:** Rashi Jain, Dhananjay Yadav, Nidhi Puranik, Randeep Guleria, Jun-O Jin

**Affiliations:** 1 Department of Pulmonary Critical Care and Sleep Medicine, AIIMS, New Delhi 110029, India; rashi473@gmail.com (R.J.); randeepguleria2002@yahoo.com (R.G.); 2 Department of Medical Biotechnology, Yeungnam University, Gyeongsan 712-749, Korea; dhanyadav16481@gmail.com; 3 Department of Biological Science, Bharathiar University, Coimbatore, Tamil Nadu-641046, India; nidhipuranik30@gmail.com; 4 Shanghai Public Health Clinical Center & Institutes of Biomedical Sciences, Shanghai Medical College, Fudan University, Shanghai 201508, China

**Keywords:** sarcoidosis, biomarkers, diagnosis, cause, management

## Abstract

Sarcoidosis is a multisystem granulomatous disease with nonspecific clinical manifestations that commonly affects the pulmonary system and other organs including the eyes, skin, liver, spleen, and lymph nodes. Sarcoidosis usually presents with persistent dry cough, eye and skin manifestations, weight loss, fatigue, night sweats, and erythema nodosum. Sarcoidosis is not influenced by sex or age, although it is more common in adults (< 50 years) of African-American or Scandinavians decent. Diagnosis can be difficult because of nonspecific symptoms and can only be verified following histopathological examination. Various factors, including infection, genetic predisposition, and environmental factors, are involved in the pathology of sarcoidosis. Exposures to insecticides, herbicides, bioaerosols, and agricultural employment are also associated with an increased risk for sarcoidosis. Due to its unknown etiology, early diagnosis and detection are difficult; however, the advent of advanced technologies, such as endobronchial ultrasound-guided biopsy, high-resolution computed tomography, magnetic resonance imaging, and 18F-fluorodeoxyglucose positron emission tomography has improved our ability to reliably diagnose this condition and accurately forecast its prognosis. This review discusses the causes and clinical features of sarcoidosis, and the improvements made in its prognosis, therapeutic management, and the recent discovery of potential biomarkers associated with the diagnostic assay used for sarcoidosis confirmation.

## 1. Introduction

Sarcoidosis is a systemic multisystem inflammatory disorder of unknown etiology characterized by the presence of non-caseating granulomas. The first case of sarcoidosis was reported in 1877 by Jonathan Hutchinson at the King’s College Hospital in London (United Kingdom) [1]. In 1889, Ernest Besnier described the cutaneous hallmarks of chronic sarcoidosis as lupus pernio. Later, Caesar Boeck used the term sarkoid (sarcoid) for the first time when he assumed that these lesions were similar to sarcoma, but benign. In India, the first case of sarcoid was published in the Journal of the School of Tropical Medicine, Calcutta in 1956, while in 1923 the first case of Familial sarcoidosis was recorded in two affected sisters [2]. 

Despite its long history, this disease remains enigmatic. Unidentified etiology and the multisystemic nature of the disease have made it more complex. Previous data suggested that at least 90% of sarcoidosis patients have manifestations in the lungs [3,4]. In addition to the lungs, the skin, liver, spleen, lymph nodes, upper respiratory tract, heart, and nervous system have all been shown to be affected by this disease and account for between 10 and 30 % [5]. Sarcoidosis occurs worldwide and has been reported in all racial and ethnic groups; no race, sex, or age is immune to it [4,6]. The clinical presentation of sarcoidosis varies depending on the specific organ involved. Sarcoidosis may present with a wide range of clinical assignations ranging from asymptomatic to fatal. The etiology of the disease is still unknown but some studies have reported that an unidentified antigen processed by activated macrophages instigates an immune response regulated by T-cells and macrophages. These activated cells discharge various mediators, including cytokines, chemokines, and reactive oxygen species that may be involved in the progression of sarcoidosis [7,8]. Many studies suggest that not only unknown antigens are responsible for this disease but also genetic susceptibility, environmental factors, and in some instances, this disease may be result from autoimmune activation [9,10]. 

To identify the studies included in this review, we performed an intensive search of the electronic databases, PubMed and MEDLINE, for relevant studies published between 1980 and the present using the following terms: sarcoidosis, pulmonary sarcoidosis, and extrapulmonary sarcoidosis. Bibliographies of all selected articles were reviewed, and we also included any relevant information from our personal files. More than 100 articles were extensively reviewed for the purpose of this review. 

## 2. Epidemiology

The prevalence and incidence of sarcoidosis are not well known worldwide owing to the challenges associated with ascertaining the number of asymptomatic patients. Sarcoidosis affects individuals of all ages irrespective of race or ethnicity, with maximum incidence among people aged 20–39 years, and quite more prevalent in women, non-smokers, and in rural communities [11]. In Europe, a higher onset of the disease has been recorded in the northern part (around 60 per 100,000) than in southern European countries, including Italy (<10 per 100,000) [12,13]. In addition, the global incidence for sarcoidosis is the highest in Sweden (64/100,000) [14], 20/100 000 in the United Kingdom [15], 4.4–6.3/100,000 in Australia [16], 10/100,000 in France, 9/100,000 in Germany, 1.4/100,000 in Spain, 7/100,000 in Greece, 1.4/100,000 in Japan. Approximately 10–14/100,000 and 35.5–64/100,000 of Caucasian and in African-Americans develop sarcoidosis, respectively [17,18,19,20]. In India, the prevalence of sarcoidosis is 10–12 cases/1000 new registrations yearly, as reported by a respiratory unit in western India and 61.2/100,000 as reported by the respiratory unit of a hospital in the capital region [21,22]. Evaluation of sarcoidosis in the Indian population is still in the very early stages, and accordingly, we can assume that its prevalence is being underreported in this region. The most common comorbidities encountered in sarcoidosis patients are hyperlipidemia, obesity, thyroid disease, diabetes, osteoporosis, coronary heart disease, asthma, hypertension, chronic renal disease, and chronic obstructive pulmonary disease (COPD) [23,24]. Sarcoidosis is also often reported in patients with certain autoimmune diseases including autoimmune thyroid disease, Sjogren’s syndrome ankylosing spondylitis [25], and systemic sclerosis [26].

## 3. Causes

The exact cause of sarcoidosis is not known. Many researchers have hypothesized the role of genetic susceptibility, environmental factors, putative antigens, and autoimmunity in the development of this disease, but no single cause has been identified to date. 

### 3.1. Genetic Factors 

Various studies suggest that genetic factors could play a crucial role in establishing the risk and clinical development of sarcoidosis [27]. Eleven sarcoidosis risk loci (BTNL2, HLA-B, HLA-DPB1, ANXA11, IL23R, SH2B3/ATXN2, IL12B, NFKB1/MANBA, FAM177B, chromosome 11q13.1, and RAB23) have been identified to date [28]. A previous study reported that familial sarcoidosis occurred in 17% of African-Americans [29], while only 1.4% of Spanish people exhibited this same risk [30]. According to A Case-Control Etiologic Sarcoidosis Study (ACCESS) the chance of developing sarcoidosis is five-fold among siblings [31]. Monozygotic siblings with sarcoidosis had an 80-fold higher risk of developing the condition, although the estimated risk of developing sarcoidosis in dizygotic twins was only seven-fold [32].

Genome wide association studies have demonstrated that several HLA and non-HLA alleles are associated with the development of this disease [33]. HLA-DRB1*0301/ DQB1*0201 [34], transforming growth factor β (TGF-β) [35], tumor necrosis factor α (TNF-α) [36], and Toll-like receptor 4 (TLR-4) [37] are all considered significant indicators for susceptibility to sarcoidosis [38,39]. 

### 3.2. Environmental Risk Factors

Various environmental factors, including exposure to wood stoves, soil, tree pollen, inorganic particulates, insecticides, and nanoparticles, have been associated with an increased risk for developing sarcoidosis. In addition to these factors, some workers, such as those involved in hardware, gardening materials, building supplies, and metal work as well as ship servicemen in the navy, fire workers, and educators, are prone to sarcoidosis [40,41,42]. It has been suggested that silica exposure also triggers the risk of sarcoidosis [43]. The underlying hypothesis for this association is that the environment is an important risk factor for the development of sarcoidosis, which has been further strengthened by reports that US World Trade Center workers exposed to the crash debris, in particular firefighters; all experienced an increased risk for developing sarcoidosis or “sarcoid-like” disease [44].

### 3.3. Infection

In addition to all of the factors mentioned above, infectious agents such as mycobacteria, have been suggested to be associated with the development of sarcoidosis, because the production of granulomas is a key factor in the immune defense response against these agents. Studies have identified numerous microbial agents as a potential eliciting agents of the immune response in sarcoidosis including *Leptospira* species, *Mycoplasma* species, herpes virus, retrovirus, *Chlamydia pneumoniae*, *Borrelia burgdorferi* [45], *Pneumocystis jirovecii* [46], *Mycobacterium (M.tb)* [47], and *Propionibacterium species* [48]. Isolation of *M.tb.* DNA, from tissue specimens collected from sarcoidosis patients, with sequences specific to mycobacterial proteins, such as ESAT-6, Kat G, and SoD A, illustrate that *Mycobacterium* is the strongest candidate for infection-mediated sarcoidosis [49,50,51]. It has been reported that patients treated with interferon α therapy for hepatitis C infection developed sarcoidosis [52,53]. A few studies have suggested that hepatitis C infection on its own could increase the risk of developing sarcoidosis. However, it seems more likely that therapy with interferon α increases interferon-γ and interleukin-2 expression, stimulating granuloma formation and thus sarcoidosis [54,55].

### 3.4. Autoimmunity 

Autoimmunity has not been studied as extensively but given the underlying pathological mechanism of sarcoidosis there is certainly potential for these conditions to play a contributing role in disease development. Although no disease-specific auto-antibodies have been observed, it has been shown that the major histocompatibility complex (MHC) class II molecules on antigen-presenting cells possess an autoantigen that is recognized by the T-cell receptor (TCR) of the responding T-cells in sarcoidosis patients [56,57]. Vimentin-derived peptides are the most plausible candidate for the activation of both T-cells and B-cells in the lung [58]. Autoimmunity presents a as a novel spectrum for sarcoidosis immunopathogenesis and may help elucidate sarcoid etiology [59,60,61]. 

Another important aspect of autoimmunity is the imbalanced gut microbiome. Gianchecchi et al. reported the associations between the presence of microbiome dysbiosis and the development of autoimmune conditions [62]. Sarcoidosis overlaps with other autoimmune diseases, including rheumatoid arthritis, autoimmune thyroid disease, Sjogren’s syndrome, and ankylosing spondylitis [63]. The role of the microbiota in these autoimmune diseases has been evaluated in previous studies and been shown to lay a significant role in their pathogenesis [64]; thus, study of the microbiome of sarcoidosis patients and its correlation with other diseases could open new avenues for investigating the underlying causes of this disease [65,66]. 

## 4. Immunopathogenesis

Many etiological agents, including infectious microbes, as well as organic and inorganic compounds, contribute to the development of sarcoidosis. These antigens are first cleared by the immune system, but this is not infallible and some undegraded antigens may remain in the cells, which can initiate an immune feedback loop. In response to this feedback loop, the antigen-presenting cells (APCs), such as dendritic cells (DCs), alveolar macrophages (AMs), and alveolar epithelial cells, produce high levels of TNF-α, and secrete interleukins-12, -15, and -18, macrophage inflammatory protein-1 (MIP-1), monocyte chemoattractant protein-1 (MCP-1), and granulocyte macrophage colony-stimulating factor (GM-CSF) [67]. These APCs also present antigens to CD4+ T-cells initiating granuloma construction, a critical feature of sarcoidosis. The growth of these granulomas establishes the primary abnormality in most cases of sarcoidosis. Sarcoid granulomas are ordered, structured masses comprised of macrophages and their derivatives, epithelioid cells, giant cells, and T-cells. 

Activated CD4+ T-cells can differentiate into two distinct subsets, namely, T helper 1 (Th1) and T helper 2 (Th2) cells, based on their cytokines profile. Th1 cells predominantly secrete interleukin-2 (IL-2) and interferon-gamma (IFN-γ), while IL-4 and IL-13 are the major secretions of Th2 cells. Resolution or maintenance of granuloma is determined by the proportion of Th1 and Th2 cells, respectively. Alveolar macrophages are activated in the Th2 milieu and stimulate fibroblast and collagen proliferation culminating in progressive fibrosis [68].

Incapacitation of Tregs is also a key feature of granuloma maintenance. It is presumed that infiltrating Tregs fail to reduce the exaggerated inflammatory response, thereby contributing to granuloma persistence and integrity. Tregs also release transforming growth factor β (TGF-β) that may contribute to fibrosis and granuloma organization [69].

Th17 and Th17.1 cells have only recently been linked to the pathogenesis of sarcoidosis [70]. These cells are recruited to the disease site and are involved in the construction of the granuloma. The balance between Th17 and Treg cells is thought to be disrupted in sarcoidosis [71] and is an important factor in its prognosis [72]. The regulation of antigen processing, antigen presentation to the APCs, and cytokine release are all controlled through genetic elements and may link the various causal factors of sarcoidosis together [73,74,75]. 

## 5. Clinical Features

Sarcoidosis is often diagnosed when aberrations are identified on a chest radiograph (up to 50% of patients) during a routine examination. Based on the presence of lung infiltration and/or lymphadenopathies on the radiograph, different stages of sarcoidosis have been described [3] (Box 1) Symptoms are usually negligible and nonspecific including cough, labored breathing, chest discomfort, dyspnea, and low-grade fever [76,77]. Systemic symptoms such as tiredness, weight reduction, and night sweats, are common. Hemoptysis is rare. Sarcoidosis may be acute, sub-acute, or chronic; however, in a majority of cases, it is entirely asymptomatic. Lofgren syndrome, where erythema nodosum and bilateral hilar adenopathy are both present, is one of the classic and acute presentation of sarcoidosis. Individuals suffering from sub-acute sarcoidosis have nonspecific signs comprising frailty, fever, weight reduction, arthralgia, and peripheral lymphadenopathy [9,78]. Chronic sarcoidosis is identified following serious persistent lung engagement, with a slow onset and a high degree of individual variability. 

The multisystemic nature of sarcoidosis leads to organ specific manifestations (Table 1). Symptoms may differ from patient to patient. According to ACCESS, 95% of patients had thoracic engagement, 50% had extra thoracic symptoms, and 2% had unaccompanied extra thoracic sarcoidosis [4]. In a study that used 18F-fluorodeoxyglucose positron emission tomography/computed tomography (18 FDG-PET/CT), the following four sarcoidosis phenotypes were identified and evaluated: thoracic nodal hilar-mediastinal, thoracic nodal hilar-mediastinal and lungs, extended thoracic and extra-thoracic only nodal phenotype including inguinal-abdominal-supraclavicular stations, and all of the above plus systemic organs and tissues such as muscles-bones-spleen and skin [79]. Most clinical studies agree that owing to the multi-organ and system granulomatous potential of sarcoidosis, a multifaceted approach is necessary to evaluate the possibility of extrapulmonary localizations of this disease.

Box 1 **Scadding’s staging of sarcoidosis.**

**Radiographic Type**

**Radiographic Characteristics**
0No visible findingsIBilateral hilar lymphadenopathyIIBilateral hilar lymphadenopathy and parenchymal infiltrationIIIParenchymal infiltration without hilar adenopathy in regular chest radiographyIVAdvanced fibrosis with severe distortion of the normal lung architecture predominately in the middle and upper lobes with evidence of bronchiectasis, hilar retraction, bulla, cysts and more rarely “honeycombing”

## 6. Screening and Diagnosis

Diagnosis of sarcoidosis always poses a challenge to clinicians. Owing to its multisystemic nature and unidentified etiology, the diagnosis of this condition can be difficult and is often delayed; however, early diagnosis is indispensable for patient management. Sarcoidosis is usually diagnosed when radiological and typical clinical data are reinforced by histological confirmation of non-necrotic granulomas. To establish any confirmed diagnosis, patients should undergo multiple clinical examinations, depending on organ involvement, as a specific diagnostic assay is still lacking (Table 2). 

Numerous imaging techniques have also been assessed for their diagnostic utility in the identification of sarcoidosis, but their utility is mostly restricted to specific organs. Despite these limitations, high-resolution computed tomography (HRCT), magnetic resonance imaging (MRI), and 18F-fluorodeoxyglucose positron emission tomography (FDG-PET) have improved the diagnosis of sarcoidosis. These techniques are equally effective in evaluating a patient’s response to treatment. 

**Figure 1:**
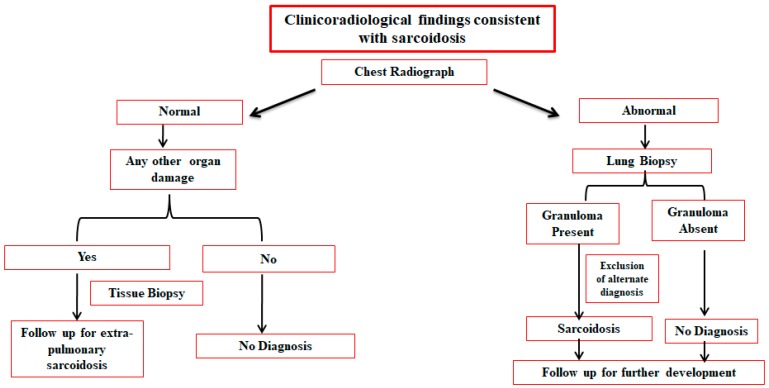
Diagnostic management of sarcoidosis.

All examinations mentioned in Table 2 can be used to identify sarcoidosis in different organs; however, no assay has been established as the gold standard. One or more tests can be performed in combination to confirm the presence of sarcoidosis, but patients’ history, symptoms, clinical signs, and particularly, expert clinical discretion always complement the finding of the relevant medical examination. Stepwise evaluation of all of the available information, excluding non-specific data, should be undertaken to confirm diagnosis (Figure 1). 

## 7. Biomarkers for Sarcoidosis

A biological marker or biomarker refers to a broad subcategory of medical signs that can be accurately, objectively, and reproducibly measured [127]. Several biomarkers have been proposed for the diagnosis of sarcoidosis and the monitoring of its progression, but none has been accepted wholly in practice [111]. The difficulty in identifying and evaluating biomarkers for sarcoidosis is linked to its dubious etiology, non-specific symptoms, and multiple disease phenotypes. Due to this lack of biomarkers, diagnosis, prognosis, treatment response, and clinical outcomes for this disease are not thoroughly predictable. 

Various biomarkers, including, serological biomarkers, bronchoalveolar lavage (BAL) biomarkers, and exhaled breath biomarkers, have been proposed numerous studies, but all have been shown to have limited applicability. Serological biomarkers should be the area of most focus for researchers moving forward as these are the least invasive and most accessible [128]. Although higher serum angiotesin converting enzyme (SACE) and BAL lymphocyte ratios are widely discussed, their utility is limited as these are not specific to sarcoidosis. In Table 3, we discuss all the plausible biomarkers for the evaluation of sarcoidosis. 

The application of biomarkers in the diagnosis and prognosis of sarcoidosis is still in its infancy with relatively few biomarkers appearing to have any real clinical application. However, the advent of “omics” type approaches (consisting of genomics, proteomics, transcriptomics, metabolomics, microbiomics, and metallomics) and the increasing number of studies applying these techniques suggest that we may soon have more valid candidates to choose from. Thus, while biomarkers are not currently a viable alternative for diagnostic applications, they may soon become effective [168].

In the previous decade, transcriptomics have identified novel gene expression profiles underlying the pathogenesis of sarcoidosis [169]. All these transcriptomic datasets validate the major role of IFN-γ-driven STAT1 signaling and type I IFN signaling in sarcoidosis [170]. Micro-RNAs have also been shown to have some potential as biomarkers for the diagnosis of sarcoidosis. miRNA-29A, hsa-miR-4306, and hsa-miR-6729-5p have been shown to be associated with sarcoidosis, acting as non-invasive biomarkers [171,172]. Metabolic changes play a crucial role in the progression of inflammation. ^1^H nuclear magnetic resonance (NMR)-based metabolomic analysis identified metabolites and metabolic pathways that can discriminate sarcoidosis patients from healthy ones. Acetoacetate, 3-hydroxybutyrate, carnitine, cystine, and trimethylamine N-oxide levels are significantly increased in sarcoidosis, with dysregulation of ketone bodies and citric cycle metabolism also being identified as hallmarks of this disease [173,174].

## 8. Treatment

In sarcoidosis, a decision on the appropriate intervention precedes the decision of whether or not to treat the patient. Not every patient needs to be treated. The decision to treat a sarcoidosis patient is predicated according to the development of specific symptoms and disease progression evidenced by worsening functional status and imaging abnormalities [175,176]. Patients can be followed-up over long periods because spontaneous resolution may occur during this time frame. Development of dangerous clinical conditions and a significant impairment in the quality of life are two major indications for clinicians to start interventional treatment [177]. Therapeutic strategies should include mental and emotional well-being, in addition to physical well-being. If treatment is to be initiated, oral corticosteroids are the first line of treatment. Corticosteroids have proved reliable in providing symptomatic relief and reversing organ dysfunction, but the risks of using corticosteroids is always a matter of concern [178].

Treatment is often initiated with 0.5–0.75 mg of prednisolone per kg (body weight) daily for 4 weeks and tapered by 10 mg every 4 weeks, depending on the disease response [179]. It is sometimes advised that the dose of 0.5-0.75 mg/kg of prednisolone is too high and doses of 20 mg of prednisone can be used as an alternative. When pulmonary function has improved, therapy can be terminated, which is usually within 6-12 months. For many patients who have mild clinical manifestations, such as skin lesions, anterior uveitis, or cough, corticosteroid treatment should be instigated. For those necessitating systemic treatment, most will recover in a reasonably short time frame but there is a small group of patients who develop chronic disorders that do not recuperate after 2–5 years. These chronic patients frequently need long-term treatment, which can necessitate the use of corticosteroids or additional therapies for more than 5 years. 

**Figure 2:**
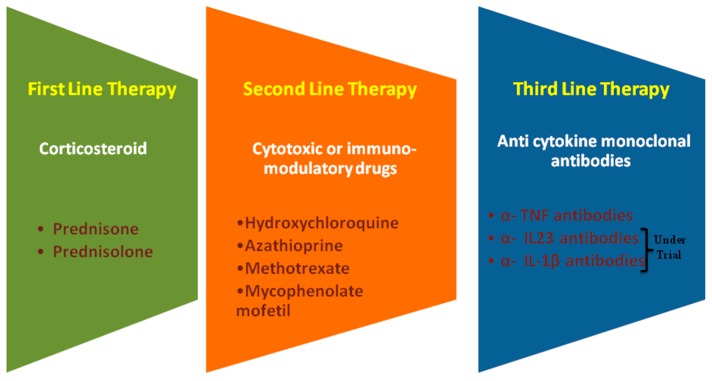
Therapeutic options for first, second, and third-line treatment of sarcoidosis.

For patients with intolerable adverse responses to steroids, corticosteroid-sparing regimens can also be administered. These are considered second line treatments and rely on therapeutics such as azathioprine [180], methotrexate [181], mycophenolate mofetil [182,183], cyclosporine [184], cyclophosphamide [185], leflunomide [186], and hydroxychloroquine [187] for symptomatic relief, but all of these drugs have been shown to be less effective than the steroid interventions (Figure 2).

Mechanism-based therapeutic treatment is the most advanced and targeted approach for the treatment of sarcoidosis. Different cytokines play a pivotal role in the immunopathogenesis of sarcoidosis. Anti-cytokine monoclonal antibodies are a specific way to modulate cytokine networks, thus influencing disease progression [188]. These cytokine-directed treatments are manifested as third line therapies. TNF-α is known to play a significant role in the formation of the granulomas associated with sarcoidosis [189]. The use of anti-TNF antibodies such as infliximab [190,191] or adalimumab [192] has shown some therapeutic benefits, although these gains have been relatively low. Recent studies describing the involvement of Th17 cells and their related cytokines in the pathogenesis of sarcoidosis, have suggested that IL-23 and IL-1β, inducers of Th17 differentiation, are useful targets for therapeutic interventions. Treatment with ustekinumab and canakinumab was recently evaluated with mixed results. Ustekinumab did not show any efficacy in pulmonary sarcoidosis and the results for canakinumab are still awaited [193] (NCT2888080). In addition, there are still relatively few guidelines for the clinical intervention of sarcoidosis [194,195,196,197] but surveillance for 3–12 months is typically endorsed to determine the overall course of the disease [198].

Personalized medicine is a novel medical doctrine focused on tailoring therapeutic management of various diseases [199]. The goal of precision medicine is to address disease prevention, diagnosis, and treatment while considering individual patient variability. Integration of different omics data presents comprehensive overviews of pathological molecular pathways that can be targeted for the development and application of precision medicine [200]. Multi-omics integrative analysis generates vast amounts of big data from sarcoidosis samples including genomic, transcriptomics, proteomic, and phenomic studies, all of which have been used to describe novel candidate regions and genes, altered in sarcoidosis [201]. These new data analysis methods are bridging the gap between conventional therapies and advanced care and are bound to open new therapeutic paradigms for this complex disease.

## 9. Conclusions 

Despite extensive research over the past several decades, the etiological agents of sarcoidosis remain unknown. Numerous potential etiological agents have been identified and the most recent hypothesis suggests that host-microbe interaction and genetic factors play an important role in the pathogenesis of this disease when they interact with various environmental factors, which results in the clinical presentation of this disease. To cure this disease, timely diagnosis is important; therefore, there is a critical need for clinicians to develop potent diagnostic tools for the identification and prognosis of sarcoidosis. Recently, new diagnostic strategies for sarcoidosis, including HRCT, FDG-PET scanning, TBNA, and EBUS technologies, have reinforced its prognosis. More focus should be concentrated on the development of non-invasive biomarkers. Big data analysis with the integration of ‘-omics’ data might elucidate the etiology and pathogenesis of sarcoidosis. Corticosteroids play an important role in the treatment of sarcoidosis, but they evoke many side effects if used for a long period. Second line and targeted treatments could be promising alternatives for the treatment of sarcoidosis in the near future. Precision medicine is the new hope in this field and should be monitored closely for progress toward targeted interventions. For better disease management, multifaceted approaches remain the best practice to ensure competent and effective patient care.

## Figures and Tables

**Table 1:** List of organs involved in sarcoidosis.

Organ Involvement	Prevalence of Organ Involvement	Manifestations	References
**Lung involvement**	more than 90% (With hilar and mediastinal lymph node)	Dry cough, wheezing, dysponea, fatigue Acute: Pleural effusion, pericardial effusion, pneumothorax, and lymph-node Chronic: lung fibrosis and respiratory failure	[80,81,82]
**Lymph node involvement**	20% of patients	Peripheral lymphadenopathy, affected lymph nodes are moderately swollen, and are usually not painful.	[83,84,85]
**Endocrine and exocrine involvement**	Thyroid glands and parotid glands are usually affected in 20%–50% of cases	Thyroid dysfunction (5%), Parotid enlargement (5%–10%), hypothalamic-pituitary effects (for example, diabetes insipidus),	[86,87]
**Skin involvement**	20%–30% of patients	Erythema nodosum (most common), profuse sweating, nodules, papules and plaques.	[88,89]
**Eye involvement**	more than 40% of patients	pain, photophobia, and hyperaemia, sometimes associated with the Löfgren syndrome	[90,91,92]
**Bone involvement**	1%–13% of patients	Osteoporosis and osteopenia are common, Nodular lesions, cystic lesions involving the joints, arthritis and arthralgia	[93,94,95]
**Upper respiratory tract**	In most patients with systemic sarcoidosis	Larynx, nasopharynx and nose are affected	[96,97,98]
**Renal involvement**	5%	Renal calculi, nephrocalcinosis, interstitial nephritis, and kidney failure	[99,100]
**Cardiac involvement**	20%–27% of sarcoidosis	Heart failure, arrhythmias, syncope	[101,102]
**Neurological involvement or neurosarcoidosis**	less than 10% of patients	Facial palsy, Meningeal inflammation, encephalopathy, vasculopathy, seizures, hydrocephalus, and mass lesions	[103,104]
**Liver and spleen involvement**	18%	Hepatosplenomegaly, intrahepatic cholestasis, and portal hypertension and altered liver function	[105,106,107]

**Table 2:** A list of conventional diagnostic tests for sarcoidosis.

Test	Indication for Sarcoidosis	References
Physical examination	fever, fatigue, malaise, weight loss, and erythema nodosum	[108]
Routine ophthalmologic examination	orbital and eyelid granulomas	[109]
Peripheral blood count	Lymphopenia	[110]
Renal function tests	High level of calcium, urea, and creatinine	[111]
Urine analysis	Hypercalciurea	[112]
Pulmonary function Tests	Assess pulmonary involvement and disease severity	[113]
Tissue biopsy	For the presence of granuloma (Lungs, lymph node, skin, salivary gland, conjunctiva)	[114]
Bronchial Biopsy	Detect pulmonary involvement, (Endobronchial ultrasound-guided transbronchial needle aspirate [EBUS-TBNA], Trans and endobronchial Biopsy)	[115,116]
Tuberculin skin test (Mantoux)	Negative in the most sarcoidosis patients	[117]
Chest X-ray	Bilateral hilar lymphadenopathy, Disseminated nodules in the lungs	[118,119]
HRCT	Differentiation of sarcoidosis from other pulmonary conditions	[120,121]
FDG-PET	Highly sensitive to detect cardiac and pulmonary involvement	[122]
Electrocardiogram (ECG)	Repolarization disturbances, Ectopic beats, Rhythm abnormalities	[123,124]
MRI	Detect neurological involvement, spinal cord, meninges, skull vault, and pituitary lesions.	[125,126]

**Table 3:** List of potential biomarkers of sarcoidosis.

Biomarkers	Indication for Sarcoidosis	Diagnostic Value	Prognostic Value	Disease Severity Assessment	References
**Serological Biomarkers**					
SACE	Indicates total granuloma load. Higher in sarcoidosis patients	+	−	++	[129,130,131]
Chitotriosidase	Produced by alveolar macrophages Increased level in sarcoidosis	−	−	++	[132,133,134]
Lysozyme	Produced by macrophages and giant epithelioid cells Higher in sarcoidosis patients	−	−	+	[135,136]
Neopterin	Produced by activated macrophages and monocytes Elevated level in sarcoidosis	−	−	+	[137,138,139]
Hypercalcemia	Higher concentration of calcium in sera of most sarcoidosis patient.	−	−	+	[140,141,142]
Soluble IL2 receptor	Marker of T cell activation Higher in sarcoidosis patients	−	+	++	[143,144,145]
SAA	Elevates the production of TNF-α, IL-18 and IL-10 in lung cells leading to T cell exhaustion Higher in sarcoidosis patients	+	−	+	[146,147,148]
Chemokines	Higher production of CCL18 led to pulmonary fibrosis High serum level of CXCL9, CXCL10 in sarcoidosis	−	+	+	[149,150,151]
KL 6	Indicates lymphocytic alveolitis and increased pulmonary Elevated level in sarcoidosis	−	+	+	[152]
IFN-gamma	Th1 inflammatory cytokine Sarcoidosis promotes IFN γ secretion	−	−	−	[153,154]
TGF-β	High TGF-β led to the development of fibrosis and chronic disease.	−	+	+	[155,156]
TNF-α	Maintenance of granuloma Higher secretion by macrophages	−	−	−	[157,158]
**Biomarkers in BAL**					
CD4/CD8 ratio in BAL	Sarcoidosis patients have a higher ratio of CD4/CD8	+	−	+	[159,160]
Percentage of White Blood cells in BAL	The high percentage of lymphocytes was observed in patients	-	−	+	[161,162]
**Exhaled Breath Biomarkers**					
8-isoprostane	Oxidative stress marker Higher in patients with sarcoidosis	+	−	−	[163,164]
Carbon monoxide	High concentration in sarcoidosis than control	−	−	−	[165]
Nitric oxide	Heterogeneity in data	−	−	−	[166,167]

SACE: Serum angiotensin converting enzyme; SAA: Serum Amyloid A; IL2: Interleukin 2; CCL18: Chemokine ligand 18; CXCL9: C-X-C Motif; Chemokine Ligand 9; CXCL10: C-X-C Motif Chemokine Ligand 10; TNF-α: Tumor Necrosis Factor-α; IL-18: Interleukin 18; IL-10: Interleukin 10; KL 6: Kerbs von Lungren 6 antigen; TGF-β: Transforming Growth Factor-β.
